# [(*E*)-2-(3,5-Dibromo-2-oxidobenzyl­ideneamino)-3-(4-hydroxy­phen­yl)propionato-κ^3^ *O*,*N*,*O*′](dimethyl­formamide-κ*O*)copper(II)

**DOI:** 10.1107/S1600536808007915

**Published:** 2008-03-29

**Authors:** Ming-Xiong Tan, Zhen-Feng Chen, Zhou Neng, Hong Liang

**Affiliations:** a College of Chemistry and Chemical Engineering, Central South University, Changsha, HuNan 410083, People’s Republic of China; b Department of Chemistry and Biology, Yu Lin Normal College, Yulin, Guangxi 537000, People’s Republic of China; c Key Laboratory of Medicinal Chemical Resources and Molecular Engineering, Ministry of Education, College of Chemistry and Chemical Engineering, Guangxi Normal University, Yucai Road 15, Guilin 541004, People’s Republic of China

## Abstract

In the title complex, [Cu(C_16_H_11_Br_2_NO_4_)(C_3_H_7_NO)]_2_, there are two unique mol­ecules in the asymmetric unit. Each Cu^II^ atom is coordinated by two O atoms and one N atom from the tridentate ligand *L* ^2−^ [*L*H_2_ = (*E*)-2-(3,5-dibromo-2-hydroxy­benzyl­idene­amino)-2-(4-hydroxy­phenyl)acetic acid] and the O atom of a dimethyl­formamide mol­ecule to give a slightly distorted square-planar geometry. The two unique mol­ecules form a dimer through weak C—H⋯O hydrogen bonds. In the dimer, the Cu⋯Cu distance is 3.712 (1) Å. In the crystal structure, mol­ecules form a one-dimensional chain through C—H⋯O hydrogen bonds. These are further aggregated into a three-dimensional network by O—H⋯O and C—H⋯O hydrogen bonds.

## Related literature

For related structures, see: Li *et al.* 2008 ▶; Zhang *et al.* (2007*a* ▶,*b* ▶). For preparative procedures, see: Xia *et al.* (2007 ▶); Liu *et al.* (2007 ▶).
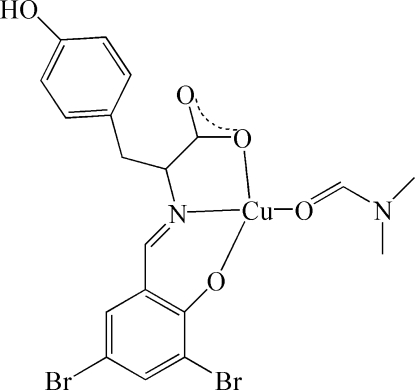


## Experimental

### 

#### Crystal data

[Cu(C_16_H_11_Br_2_NO_4_)(C_3_H_7_NO)]
*M* *_r_* = 577.71
Triclinic, 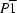

*a* = 11.4316 (19) Å
*b* = 11.840 (2) Å
*c* = 15.984 (2) Å
α = 88.998 (3)°
β = 83.562 (2)°
γ = 73.210 (2)°
*V* = 2057.9 (6) Å^3^
*Z* = 4
Mo *K*α radiation
μ = 4.98 mm^−1^
*T* = 298 (2) K
0.33 × 0.18 × 0.14 mm

#### Data collection

Bruker SMART CCD area-detector diffractometer
Absorption correction: multi-scan (*SADABS*; Sheldrick,1996 ▶) *T*_min_ = 0.267, *T*_max_ = 0.498
10763 measured reflections
7143 independent reflections
3697 reflections with > 2s(*I*)
*R*_int_ = 0.041

#### Refinement

*R*[*F* ^2^ > 2σ(*F* ^2^)] = 0.050
*wR*(*F* ^2^) = 0.093
*S* = 1.00
7143 reflections
523 parameters
H-atom parameters constrained
Δρ_max_ = 0.60 e Å^−3^
Δρ_min_ = −0.55 e Å^−3^

### 

Data collection: *SMART* (Bruker, 2001 ▶); cell refinement: *SAINT* (Bruker, 2001 ▶); data reduction: *SAINT*; program(s) used to solve structure: *SHELXS97* (Sheldrick, 2008 ▶); program(s) used to refine structure: *SHELXL97* (Sheldrick, 2008 ▶); molecular graphics: *SHELXTL* (Sheldrick, 2008 ▶); software used to prepare material for publication: *SHELXTL*.

## Supplementary Material

Crystal structure: contains datablocks global, I. DOI: 10.1107/S1600536808007915/sj2468sup1.cif

Structure factors: contains datablocks I. DOI: 10.1107/S1600536808007915/sj2468Isup2.hkl

Additional supplementary materials:  crystallographic information; 3D view; checkCIF report

## Figures and Tables

**Table:** 

Cu1—O4	1.874 (4)
Cu1—N1	1.893 (5)
Cu1—O5	1.917 (5)
Cu1—O1	1.932 (4)
Cu2—O9	1.874 (4)
Cu2—N3	1.907 (5)
Cu2—O6	1.922 (4)
Cu2—O10	1.932 (5)

**Table:** 

O4—Cu1—N1	94.3 (2)
O4—Cu1—O5	92.5 (2)
N1—Cu1—O5	173.1 (2)
O4—Cu1—O1	177.0 (2)
N1—Cu1—O1	84.6 (2)
O5—Cu1—O1	88.5 (2)
O9—Cu2—N3	95.0 (2)
O9—Cu2—O6	178.9 (2)
N3—Cu2—O6	85.1 (2)
O9—Cu2—O10	90.9 (2)
N3—Cu2—O10	173.7 (2)
O6—Cu2—O10	89.0 (2)

**Table 2:** Hydrogen-bond geometry (Å, °)

*D*—H⋯*A*	*D*—H	H⋯*A*	*D*⋯*A*	*D*—H⋯*A*
C18—H18*C*⋯O7	0.96	2.59	3.364 (9)	137
C37—H37*C*⋯O2	0.96	2.48	3.307 (8)	144
O3—H3⋯O1^i^	0.82	1.98	2.772 (6)	163
O8—H8⋯O6^ii^	0.82	2.07	2.888 (6)	176
C16—H16⋯O7^iii^	0.93	2.52	3.422 (9)	163
C29—H29⋯O2^iv^	0.93	2.45	3.291 (8)	150
C35—H35⋯O2^iv^	0.93	2.59	3.408 (8)	147

Symmetry codes: (i) 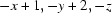; (ii) 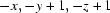; (iii) 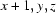; (iv) 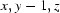.

**Fig. 1.**
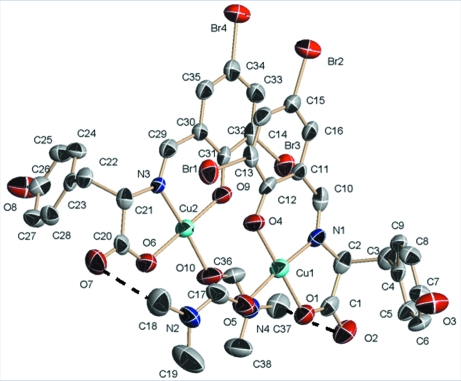
The asymmetric unit of (I), with 30% probability displacement ellipsoids for non-H atoms. Hydrogen atoms have been omitted and C—H···O hydrogen bonds are drawn as dashed lines.

**Fig. 2.**
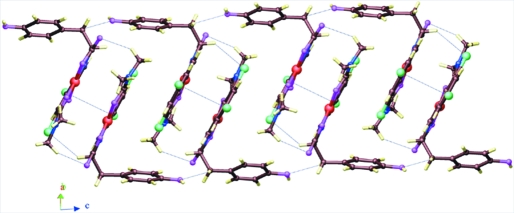
The formation of one-dimensional chains along c. Hydrogen bonds are drawn as dashed lines.

**Fig. 3.**
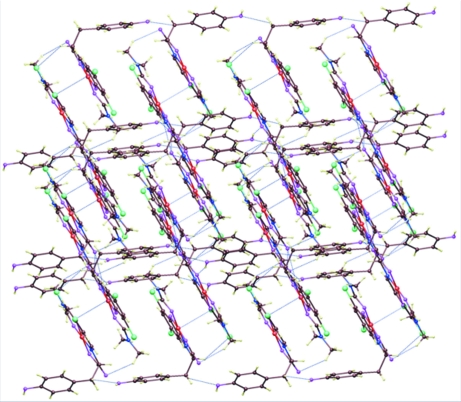
Crystal packing of (I) showing the three-dimensional network, with hydrogen bonds drawn as dashed lines.

## supplementary crystallographic information

### Comment

Herein, we report the structure of a new mononuclear copper coordination complex [Cu(*L*)(C_3_H_7_NO)]_2_ (1), Fig. 1, of the chiral ligand (*E*)-2-(3,5-dibromo-2-oxidobenzylideneamino)-2-(4-hydroxyphenyl)acetate *L*H_2_. The Cu(II) atom coordinates a dimethylformamide molecule and the tridentate anionic ligand *L*^2-^ which binds through the N atom and carboxylate and phenolate O atoms. Although the *L*H_2_ ligand is chiral, the compound crystallizes as a racemate with two molecules in the asymmetric unit. The coordination geometry about each copper atom is slightly distorted square planar, Table 1. A s expected all other bond distances and angles are within normal ranges (Zhang *et al.*, 2007*a*,b).

The two unique molecules form a dimer through weak C18—H18C···O7 and C37—H37C···O2 hydrogen bonds, Table 2. In the dimer, the Cu1···Cu2 distance is 3.712 (1) Å. In the crystal structure, molecules of (I) form a one-dimensional chain along c (Fig. 2) through C3–H3B···O8 and C22–H22B···O3 hydrogen bonds. These chains then form a three-dimensional network through O3—H3···O1 and O8—H8···O6 hydrogen bonds and C29–H29···O2 and C35—H35···O2 interactions (Table 2, Figure 3).

### Experimental

Complex (I) was prepared following the procedure described by Liu *et al.* (2007) and Xia *et al.* (2007) as follows. 3,5-Dibromo-2-hydroxy-benzaldehyde(0.560 g, 2.0 mmol) and 4-hydroxyl-phenylalanine (0.3624 g, 2.0 mmol) were dissolved in 10 ml absolute methanol. The mixture was stirred for 1 h at room temperature to give a yellow solution. 2 ml DMF and 10 ml of a methanolic solution of CuSO_4_.5H_2_O (0.5 g, 2 mmol) were added, the mixture was refluxed for another 1 h at 363 K, and the resulting blue solution was filtered. Blue single crystals suitable for *X*–ray analysis were obtained by slow evaporation of the filtrate at room temperature. Yield: 80.1% (based on copper). Elemental analysis for [Cu(C_16_H_11_Br_2_NO_4_)(C_3_H_7_NO)]_2_ calculated: C 46.69, H 3.71, N 5.73%; found: C 46.65, H 3.81, N 5.71%.

### Refinement

All H-atoms were positioned geometrically and refined using a riding model with d(C—H) = 0.93 Å, *U*_iso_=1.2*U*_eq_ (C) for aromatic 0.96 Å, *U*_iso_ = 1.5*U*_eq_ (C) for CH_3_ atoms and 0.82 Å, *U*_iso_ = 1.5*U*_eq_ (O) for the OH groups.

### Crystal data

**Table:**

[Cu(C_16_H_11_Br_2_NO_4_)(C_3_H_7_NO)]	*Z* = 4
*M**_r_* = 577.71	*F*_000_ = 1140
Triclinic, *P*1	*D*_x_ = 1.865 Mg m^−^^3^
Hall symbol: -P 1	Mo *K*α radiation λ = 0.71073 Å
*a* = 11.4316 (19) Å	Cell parameters from 1919 reflections
*b* = 11.840 (2) Å	θ = 2.2–21.6º
*c* = 15.984 (2) Å	µ = 4.98 mm^−^^1^
α = 88.998 (3)º	*T* = 298 (2) K
β = 83.562 (2)º	Prism, blue
γ = 73.210 (2)º	0.33 × 0.18 × 0.14 mm
*V* = 2057.9 (6) Å^3^	

### Data collection

**Table:**

Bruker SMART CCD area-detector diffractometer	7143 independent reflections
Radiation source: fine-focus sealed tube	3697 reflections with > 2s(*I*)
Monochromator: graphite	*R*_int_ = 0.041
*T* = 298(2) K	θ_max_ = 25.0º
φ and ω scans	θ_min_ = 1.3º
Absorption correction: multi-scan(SADABS; Sheldrick,1996)	*h* = −13→13
*T*_min_ = 0.267, *T*_max_ = 0.498	*k* = −14→10
10763 measured reflections	*l* = −19→16

### Refinement

**Table:**

Refinement on *F*^2^	Secondary atom site location: difference Fourier map
Least-squares matrix: full	Hydrogen site location: inferred from neighbouring sites
*R*[*F*^2^ > 2σ(*F*^2^)] = 0.050	H-atom parameters constrained
*wR*(*F*^2^) = 0.093	*w* = 1/[σ^2^(*F*_o_^2^) + (0.024*P*)^2^] where *P* = (*F*_o_^2^ + 2*F*_c_^2^)/3
*S* = 1.00	(Δ/σ)_max_ < 0.001
7143 reflections	Δρ_max_ = 0.60 e Å^−^^3^
523 parameters	Δρ_min_ = −0.55 e Å^−^^3^
Primary atom site location: structure-invariant direct methods	Extinction correction: none

### Special details

**Table:**

Geometry. All e.s.d.'s (except the e.s.d. in the dihedral angle between two l.s. planes) are estimated using the full covariance matrix. The cell e.s.d.'s are taken into account individually in the estimation of e.s.d.'s in distances, angles and torsion angles; correlations between e.s.d.'s in cell parameters are only used when they are defined by crystal symmetry. An approximate (isotropic) treatment of cell e.s.d.'s is used for estimating e.s.d.'s involving l.s. planes.
Refinement. Refinement of F^2^ against ALL reflections. The weighted R-factor wR and goodness of fit S are based on F^2^, conventional R-factors R are based on F, with F set to zero for negative F^2^. The threshold expression of F^2^ > σ(F^2^) is used only for calculating R-factors(gt) etc. and is not relevant to the choice of reflections for refinement. R-factors based on F^2^ are statistically about twice as large as those based on F, and R-factors based on ALL data will be even larger.

### Fractional atomic coordinates and isotropic or equivalent isotropic displacement parameters (Å^2^)

**Table:**

	*x*	*y*	*z*	*U*_iso_*/*U*_eq_	
Cu1	0.45708 (7)	0.70847 (7)	0.17285 (5)	0.0437 (2)	
Cu2	0.33188 (7)	0.52131 (7)	0.32557 (5)	0.0433 (2)	
Br1	0.47185 (6)	0.32932 (7)	0.06179 (5)	0.0569 (2)	
Br2	0.95715 (7)	0.15518 (7)	0.14381 (6)	0.0859 (3)	
Br3	0.70303 (6)	0.51591 (6)	0.44153 (5)	0.0583 (2)	
Br4	0.88589 (7)	0.02902 (7)	0.36347 (6)	0.0810 (3)	
N1	0.6098 (5)	0.6878 (5)	0.2154 (3)	0.0386 (14)	
N2	0.1415 (5)	0.6973 (6)	0.0891 (4)	0.0586 (17)	
N3	0.3513 (4)	0.3622 (4)	0.2930 (3)	0.0312 (13)	
N4	0.3335 (5)	0.8409 (5)	0.4121 (3)	0.0494 (15)	
O1	0.4266 (4)	0.8725 (4)	0.2033 (3)	0.0507 (12)	
O2	0.4951 (4)	0.9914 (4)	0.2783 (3)	0.0625 (14)	
O3	0.7098 (4)	0.9130 (4)	−0.1456 (3)	0.0760 (16)	
H3	0.6752	0.9822	−0.1549	0.114*	
O4	0.4861 (4)	0.5476 (4)	0.1493 (3)	0.0492 (12)	
O5	0.2972 (4)	0.7485 (4)	0.1347 (3)	0.0592 (14)	
O6	0.1695 (4)	0.5527 (4)	0.2919 (3)	0.0509 (13)	
O7	0.0475 (4)	0.4692 (4)	0.2348 (3)	0.0709 (16)	
O8	0.0751 (4)	0.2943 (4)	0.6592 (3)	0.0816 (17)	
H8	0.0063	0.3400	0.6711	0.122*	
O9	0.4890 (4)	0.4905 (4)	0.3606 (3)	0.0431 (12)	
O10	0.2976 (4)	0.6874 (4)	0.3510 (3)	0.0645 (15)	
C1	0.5074 (7)	0.8940 (6)	0.2447 (4)	0.0443 (18)	
C2	0.6249 (6)	0.7964 (6)	0.2492 (4)	0.0452 (18)	
H2	0.6391	0.7842	0.3084	0.054*	
C3	0.7327 (6)	0.8309 (6)	0.2023 (4)	0.0483 (18)	
H3A	0.7399	0.9013	0.2289	0.058*	
H3B	0.8077	0.7682	0.2079	0.058*	
C4	0.7220 (5)	0.8543 (6)	0.1097 (4)	0.0390 (17)	
C5	0.6648 (6)	0.9636 (6)	0.0814 (4)	0.0467 (18)	
H5	0.6290	1.0245	0.1204	0.056*	
C6	0.6584 (6)	0.9870 (6)	−0.0033 (4)	0.0492 (19)	
H6	0.6202	1.0627	−0.0208	0.059*	
C7	0.7089 (6)	0.8970 (6)	−0.0613 (5)	0.0450 (18)	
C8	0.7654 (5)	0.7877 (6)	−0.0346 (4)	0.0458 (18)	
H8A	0.8003	0.7271	−0.0740	0.055*	
C9	0.7724 (5)	0.7642 (6)	0.0503 (4)	0.0394 (17)	
H9	0.8107	0.6883	0.0674	0.047*	
C10	0.6954 (6)	0.5907 (7)	0.2170 (4)	0.0427 (19)	
H10	0.7636	0.5921	0.2432	0.051*	
C11	0.6958 (6)	0.4798 (6)	0.1821 (4)	0.0339 (16)	
C12	0.5917 (6)	0.4666 (6)	0.1476 (4)	0.0384 (17)	
C13	0.6066 (6)	0.3563 (7)	0.1109 (4)	0.0434 (18)	
C14	0.7127 (6)	0.2650 (6)	0.1096 (4)	0.0464 (18)	
H14	0.7184	0.1930	0.0845	0.056*	
C15	0.8119 (6)	0.2807 (6)	0.1461 (4)	0.0473 (19)	
C16	0.8028 (6)	0.3859 (6)	0.1807 (4)	0.0451 (19)	
H16	0.8697	0.3964	0.2043	0.054*	
C17	0.2547 (7)	0.6760 (7)	0.1048 (5)	0.058 (2)	
H17	0.3072	0.6007	0.0926	0.070*	
C18	0.0959 (7)	0.6084 (7)	0.0547 (5)	0.083 (3)	
H18A	0.1634	0.5397	0.0388	0.125*	
H18B	0.0554	0.6387	0.0061	0.125*	
H18C	0.0387	0.5877	0.0964	0.125*	
C19	0.0575 (7)	0.8125 (8)	0.1092 (6)	0.117 (4)	
H19A	0.0213	0.8142	0.1666	0.175*	
H19B	−0.0061	0.8291	0.0724	0.175*	
H19C	0.1011	0.8707	0.1021	0.175*	
C20	0.1454 (6)	0.4640 (7)	0.2619 (4)	0.0453 (19)	
C21	0.2437 (5)	0.3457 (5)	0.2600 (4)	0.0390 (17)	
H21	0.2675	0.3202	0.2010	0.047*	
C22	0.1966 (5)	0.2501 (5)	0.3067 (4)	0.0427 (17)	
H22A	0.1238	0.2452	0.2829	0.051*	
H22B	0.2588	0.1748	0.2964	0.051*	
C23	0.1652 (6)	0.2694 (5)	0.4012 (4)	0.0349 (16)	
C24	0.2473 (6)	0.2088 (5)	0.4556 (5)	0.0445 (18)	
H24	0.3246	0.1613	0.4342	0.053*	
C25	0.2147 (6)	0.2189 (6)	0.5402 (5)	0.0504 (19)	
H25	0.2707	0.1775	0.5759	0.060*	
C26	0.1029 (7)	0.2877 (6)	0.5744 (5)	0.0484 (19)	
C27	0.0216 (6)	0.3516 (6)	0.5208 (4)	0.0488 (19)	
H27	−0.0538	0.4022	0.5425	0.059*	
C28	0.0536 (6)	0.3397 (6)	0.4346 (4)	0.0422 (18)	
H28	−0.0023	0.3805	0.3987	0.051*	
C29	0.4494 (6)	0.2781 (6)	0.2933 (4)	0.0387 (17)	
H29	0.4508	0.2063	0.2698	0.046*	
C30	0.5595 (6)	0.2846 (6)	0.3275 (4)	0.0354 (16)	
C31	0.5709 (6)	0.3894 (6)	0.3605 (4)	0.0353 (16)	
C32	0.6824 (6)	0.3771 (6)	0.3959 (4)	0.0431 (18)	
C33	0.7748 (6)	0.2735 (7)	0.3965 (4)	0.050 (2)	
H33	0.8461	0.2706	0.4205	0.060*	
C34	0.7605 (6)	0.1727 (6)	0.3609 (4)	0.0479 (19)	
C35	0.6547 (6)	0.1792 (6)	0.3273 (4)	0.0418 (18)	
H35	0.6453	0.1116	0.3036	0.050*	
C36	0.3602 (6)	0.7288 (6)	0.3940 (4)	0.0496 (19)	
H36	0.4290	0.6775	0.4141	0.059*	
C37	0.4104 (6)	0.8861 (6)	0.4608 (4)	0.063 (2)	
H37A	0.4721	0.8213	0.4812	0.095*	
H37B	0.3607	0.9322	0.5077	0.095*	
H37C	0.4494	0.9346	0.4259	0.095*	
C38	0.2263 (6)	0.9238 (6)	0.3835 (5)	0.075 (2)	
H38A	0.1738	0.8816	0.3644	0.112*	
H38B	0.2514	0.9687	0.3380	0.112*	
H38C	0.1823	0.9761	0.4291	0.112*	

### Atomic displacement parameters (Å^2^)

**Table:**

	*U*^11^	*U*^22^	*U*^33^	*U*^12^	*U*^13^	*U*^23^
Cu1	0.0467 (5)	0.0381 (5)	0.0484 (6)	−0.0140 (4)	−0.0101 (4)	0.0016 (4)
Cu2	0.0440 (5)	0.0341 (5)	0.0536 (6)	−0.0120 (4)	−0.0116 (4)	0.0022 (4)
Br1	0.0556 (5)	0.0620 (6)	0.0605 (6)	−0.0290 (4)	−0.0039 (4)	−0.0116 (4)
Br2	0.0567 (5)	0.0566 (6)	0.1299 (9)	0.0036 (5)	−0.0036 (5)	0.0072 (5)
Br3	0.0577 (5)	0.0530 (5)	0.0742 (6)	−0.0261 (4)	−0.0222 (4)	0.0004 (4)
Br4	0.0546 (5)	0.0557 (6)	0.1204 (8)	0.0037 (5)	−0.0123 (5)	0.0119 (5)
N1	0.048 (4)	0.032 (4)	0.039 (4)	−0.015 (3)	−0.009 (3)	0.008 (3)
N2	0.037 (4)	0.065 (5)	0.074 (5)	−0.012 (4)	−0.016 (3)	0.002 (4)
N3	0.032 (3)	0.026 (3)	0.039 (4)	−0.012 (3)	−0.006 (3)	0.002 (2)
N4	0.056 (4)	0.044 (4)	0.048 (4)	−0.016 (4)	0.002 (3)	−0.003 (3)
O1	0.055 (3)	0.042 (3)	0.058 (3)	−0.017 (3)	−0.009 (3)	0.003 (2)
O2	0.084 (4)	0.044 (3)	0.065 (4)	−0.027 (3)	−0.008 (3)	−0.013 (3)
O3	0.108 (4)	0.059 (4)	0.041 (4)	0.007 (3)	−0.005 (3)	−0.003 (3)
O4	0.046 (3)	0.035 (3)	0.066 (3)	−0.007 (3)	−0.017 (2)	0.004 (2)
O5	0.050 (3)	0.055 (4)	0.073 (4)	−0.013 (3)	−0.014 (3)	−0.005 (3)
O6	0.046 (3)	0.042 (3)	0.066 (4)	−0.011 (3)	−0.015 (2)	0.004 (2)
O7	0.049 (3)	0.086 (4)	0.087 (4)	−0.027 (3)	−0.026 (3)	0.019 (3)
O8	0.098 (4)	0.078 (4)	0.041 (4)	0.017 (3)	−0.007 (3)	0.001 (3)
O9	0.048 (3)	0.031 (3)	0.051 (3)	−0.010 (2)	−0.008 (2)	0.001 (2)
O10	0.064 (3)	0.035 (3)	0.095 (4)	−0.005 (3)	−0.033 (3)	−0.002 (3)
C1	0.061 (5)	0.038 (5)	0.037 (5)	−0.021 (5)	−0.003 (4)	0.010 (4)
C2	0.064 (5)	0.049 (5)	0.035 (5)	−0.034 (4)	−0.014 (4)	0.004 (3)
C3	0.059 (5)	0.051 (5)	0.045 (5)	−0.028 (4)	−0.018 (4)	0.000 (3)
C4	0.039 (4)	0.041 (5)	0.046 (5)	−0.024 (4)	−0.007 (4)	−0.003 (4)
C5	0.072 (5)	0.032 (5)	0.037 (5)	−0.018 (4)	0.005 (4)	−0.006 (3)
C6	0.070 (5)	0.025 (4)	0.048 (5)	−0.008 (4)	−0.003 (4)	0.001 (4)
C7	0.046 (4)	0.044 (5)	0.041 (5)	−0.007 (4)	−0.002 (4)	−0.006 (4)
C8	0.045 (4)	0.044 (5)	0.044 (5)	−0.005 (4)	−0.006 (4)	−0.013 (4)
C9	0.038 (4)	0.033 (4)	0.053 (5)	−0.015 (4)	−0.014 (4)	0.002 (4)
C10	0.051 (5)	0.065 (6)	0.027 (4)	−0.036 (5)	−0.014 (4)	0.015 (4)
C11	0.037 (4)	0.032 (4)	0.038 (4)	−0.015 (4)	−0.013 (3)	0.009 (3)
C12	0.054 (5)	0.024 (4)	0.042 (5)	−0.018 (4)	−0.005 (4)	0.007 (3)
C13	0.037 (4)	0.058 (5)	0.043 (5)	−0.025 (4)	−0.006 (3)	0.008 (4)
C14	0.054 (5)	0.030 (5)	0.055 (5)	−0.016 (4)	0.006 (4)	−0.003 (3)
C15	0.045 (5)	0.036 (5)	0.061 (5)	−0.015 (4)	−0.003 (4)	0.012 (4)
C16	0.049 (5)	0.038 (5)	0.054 (5)	−0.022 (4)	−0.006 (4)	0.013 (4)
C17	0.044 (5)	0.067 (6)	0.058 (6)	−0.005 (5)	−0.011 (4)	0.014 (4)
C18	0.076 (6)	0.092 (7)	0.097 (8)	−0.038 (6)	−0.037 (5)	0.019 (5)
C19	0.059 (6)	0.116 (9)	0.169 (11)	−0.004 (6)	−0.033 (6)	−0.046 (7)
C20	0.035 (4)	0.055 (6)	0.046 (5)	−0.016 (5)	0.000 (4)	0.021 (4)
C21	0.042 (4)	0.049 (5)	0.034 (4)	−0.023 (4)	−0.011 (3)	−0.002 (3)
C22	0.045 (4)	0.040 (4)	0.051 (5)	−0.023 (4)	−0.011 (3)	−0.004 (3)
C23	0.039 (4)	0.029 (4)	0.044 (5)	−0.021 (4)	−0.005 (4)	−0.001 (3)
C24	0.037 (4)	0.034 (4)	0.058 (6)	−0.003 (4)	−0.007 (4)	−0.002 (4)
C25	0.050 (5)	0.043 (5)	0.054 (6)	−0.002 (4)	−0.017 (4)	0.005 (4)
C26	0.061 (5)	0.042 (5)	0.042 (5)	−0.014 (4)	−0.010 (4)	0.001 (4)
C27	0.041 (4)	0.050 (5)	0.048 (5)	−0.001 (4)	−0.009 (4)	0.008 (4)
C28	0.035 (4)	0.054 (5)	0.041 (5)	−0.014 (4)	−0.017 (4)	0.011 (4)
C29	0.049 (5)	0.033 (5)	0.035 (4)	−0.015 (4)	0.002 (4)	−0.007 (3)
C30	0.033 (4)	0.035 (4)	0.040 (4)	−0.013 (4)	−0.005 (3)	0.005 (3)
C31	0.039 (4)	0.036 (5)	0.038 (4)	−0.023 (4)	−0.001 (3)	0.002 (3)
C32	0.043 (4)	0.057 (5)	0.041 (5)	−0.033 (4)	−0.005 (4)	0.002 (4)
C33	0.039 (4)	0.051 (5)	0.062 (5)	−0.014 (4)	−0.013 (4)	0.014 (4)
C34	0.045 (5)	0.050 (5)	0.051 (5)	−0.021 (4)	0.001 (4)	0.008 (4)
C35	0.050 (5)	0.037 (5)	0.040 (5)	−0.017 (4)	0.004 (4)	−0.001 (3)
C36	0.057 (5)	0.030 (5)	0.057 (5)	−0.003 (4)	−0.011 (4)	0.006 (4)
C37	0.079 (6)	0.062 (5)	0.052 (5)	−0.027 (5)	−0.001 (4)	−0.013 (4)
C38	0.061 (5)	0.051 (6)	0.103 (7)	0.004 (5)	−0.024 (5)	−0.013 (5)

### Geometric parameters (Å, °)

**Table:**

Cu1—O4	1.874 (4)	C10—C11	1.435 (8)
Cu1—N1	1.893 (5)	C10—H10	0.9300
Cu1—O5	1.917 (5)	C11—C16	1.393 (8)
Cu1—O1	1.932 (4)	C11—C12	1.415 (8)
Cu2—O9	1.874 (4)	C12—C13	1.398 (9)
Cu2—N3	1.907 (5)	C13—C14	1.371 (8)
Cu2—O6	1.922 (4)	C14—C15	1.391 (8)
Cu2—O10	1.932 (5)	C14—H14	0.9300
Br1—C13	1.912 (6)	C15—C16	1.344 (9)
Br2—C15	1.878 (7)	C16—H16	0.9300
Br3—C32	1.896 (6)	C17—H17	0.9300
Br4—C34	1.884 (7)	C18—H18A	0.9600
N1—C10	1.279 (7)	C18—H18B	0.9600
N1—C2	1.465 (7)	C18—H18C	0.9600
N2—C17	1.297 (8)	C19—H19A	0.9600
N2—C19	1.440 (9)	C19—H19B	0.9600
N2—C18	1.443 (8)	C19—H19C	0.9600
N3—C29	1.267 (6)	C20—C21	1.520 (8)
N3—C21	1.454 (6)	C21—C22	1.536 (7)
N4—C36	1.303 (8)	C21—H21	0.9800
N4—C38	1.446 (7)	C22—C23	1.518 (8)
N4—C37	1.451 (7)	C22—H22A	0.9700
O1—C1	1.278 (7)	C22—H22B	0.9700
O2—C1	1.245 (7)	C23—C28	1.362 (8)
O3—C7	1.358 (7)	C23—C24	1.387 (7)
O3—H3	0.8200	C24—C25	1.360 (8)
O4—C12	1.305 (7)	C24—H24	0.9300
O5—C17	1.228 (8)	C25—C26	1.361 (8)
O6—C20	1.274 (8)	C25—H25	0.9300
O7—C20	1.229 (7)	C26—C27	1.386 (8)
O8—C26	1.355 (7)	C27—C28	1.384 (8)
O8—H8	0.8200	C27—H27	0.9300
O9—C31	1.289 (6)	C28—H28	0.9300
O10—C36	1.245 (7)	C29—C30	1.449 (7)
C1—C2	1.506 (9)	C29—H29	0.9300
C2—C3	1.524 (8)	C30—C35	1.399 (8)
C2—H2	0.9800	C30—C31	1.403 (8)
C3—C4	1.513 (8)	C31—C32	1.421 (8)
C3—H3A	0.9700	C32—C33	1.368 (8)
C3—H3B	0.9700	C33—C34	1.391 (8)
C4—C5	1.366 (8)	C33—H33	0.9300
C4—C9	1.389 (8)	C34—C35	1.359 (8)
C5—C6	1.383 (8)	C35—H35	0.9300
C5—H5	0.9300	C36—H36	0.9300
C6—C7	1.373 (9)	C37—H37A	0.9600
C6—H6	0.9300	C37—H37B	0.9600
C7—C8	1.354 (8)	C37—H37C	0.9600
C8—C9	1.386 (8)	C38—H38A	0.9600
C8—H8A	0.9300	C38—H38B	0.9600
C9—H9	0.9300	C38—H38C	0.9600
			
O4—Cu1—N1	94.3 (2)	C11—C16—H16	119.1
O4—Cu1—O5	92.5 (2)	O5—C17—N2	124.5 (8)
N1—Cu1—O5	173.1 (2)	O5—C17—H17	117.8
O4—Cu1—O1	177.0 (2)	N2—C17—H17	117.8
N1—Cu1—O1	84.6 (2)	N2—C18—H18A	109.5
O5—Cu1—O1	88.5 (2)	N2—C18—H18B	109.5
O9—Cu2—N3	95.0 (2)	H18A—C18—H18B	109.5
O9—Cu2—O6	178.9 (2)	N2—C18—H18C	109.5
N3—Cu2—O6	85.1 (2)	H18A—C18—H18C	109.5
O9—Cu2—O10	90.9 (2)	H18B—C18—H18C	109.5
N3—Cu2—O10	173.7 (2)	N2—C19—H19A	109.5
O6—Cu2—O10	89.0 (2)	N2—C19—H19B	109.5
C10—N1—C2	120.1 (5)	H19A—C19—H19B	109.5
C10—N1—Cu1	126.0 (4)	N2—C19—H19C	109.5
C2—N1—Cu1	113.9 (4)	H19A—C19—H19C	109.5
C17—N2—C19	118.9 (7)	H19B—C19—H19C	109.5
C17—N2—C18	122.2 (7)	O7—C20—O6	123.6 (7)
C19—N2—C18	118.9 (6)	O7—C20—C21	118.7 (7)
C29—N3—C21	121.2 (5)	O6—C20—C21	117.7 (6)
C29—N3—Cu2	125.2 (4)	N3—C21—C20	108.7 (5)
C21—N3—Cu2	113.5 (4)	N3—C21—C22	112.6 (4)
C36—N4—C38	120.5 (6)	C20—C21—C22	112.3 (5)
C36—N4—C37	121.2 (6)	N3—C21—H21	107.7
C38—N4—C37	118.3 (6)	C20—C21—H21	107.7
C1—O1—Cu1	114.8 (4)	C22—C21—H21	107.7
C7—O3—H3	109.5	C23—C22—C21	115.3 (5)
C12—O4—Cu1	126.0 (4)	C23—C22—H22A	108.4
C17—O5—Cu1	123.4 (5)	C21—C22—H22A	108.4
C20—O6—Cu2	115.0 (4)	C23—C22—H22B	108.4
C26—O8—H8	109.5	C21—C22—H22B	108.4
C31—O9—Cu2	126.7 (4)	H22A—C22—H22B	107.5
C36—O10—Cu2	124.0 (4)	C28—C23—C24	118.5 (6)
O2—C1—O1	123.5 (7)	C28—C23—C22	121.2 (5)
O2—C1—C2	119.3 (7)	C24—C23—C22	120.1 (6)
O1—C1—C2	117.1 (6)	C25—C24—C23	119.9 (6)
N1—C2—C1	108.5 (6)	C25—C24—H24	120.0
N1—C2—C3	112.6 (6)	C23—C24—H24	120.0
C1—C2—C3	110.1 (5)	C24—C25—C26	122.1 (6)
N1—C2—H2	108.5	C24—C25—H25	118.9
C1—C2—H2	108.5	C26—C25—H25	118.9
C3—C2—H2	108.5	O8—C26—C25	119.7 (6)
C4—C3—C2	114.2 (5)	O8—C26—C27	121.8 (7)
C4—C3—H3A	108.7	C25—C26—C27	118.4 (7)
C2—C3—H3A	108.7	C28—C27—C26	119.5 (7)
C4—C3—H3B	108.7	C28—C27—H27	120.3
C2—C3—H3B	108.7	C26—C27—H27	120.3
H3A—C3—H3B	107.6	C23—C28—C27	121.4 (6)
C5—C4—C9	117.7 (6)	C23—C28—H28	119.3
C5—C4—C3	121.8 (6)	C27—C28—H28	119.3
C9—C4—C3	120.4 (6)	N3—C29—C30	125.3 (6)
C4—C5—C6	122.3 (6)	N3—C29—H29	117.4
C4—C5—H5	118.8	C30—C29—H29	117.4
C6—C5—H5	118.8	C35—C30—C31	121.0 (6)
C7—C6—C5	119.2 (6)	C35—C30—C29	116.3 (6)
C7—C6—H6	120.4	C31—C30—C29	122.7 (6)
C5—C6—H6	120.4	O9—C31—C30	124.6 (6)
C8—C7—O3	117.4 (6)	O9—C31—C32	120.7 (6)
C8—C7—C6	119.5 (7)	C30—C31—C32	114.7 (6)
O3—C7—C6	123.0 (6)	C33—C32—C31	124.1 (6)
C7—C8—C9	121.4 (6)	C33—C32—Br3	119.3 (5)
C7—C8—H8A	119.3	C31—C32—Br3	116.5 (5)
C9—C8—H8A	119.3	C32—C33—C34	119.0 (6)
C8—C9—C4	119.9 (6)	C32—C33—H33	120.5
C8—C9—H9	120.1	C34—C33—H33	120.5
C4—C9—H9	120.1	C35—C34—C33	119.2 (7)
N1—C10—C11	126.0 (6)	C35—C34—Br4	121.5 (6)
N1—C10—H10	117.0	C33—C34—Br4	119.3 (5)
C11—C10—H10	117.0	C34—C35—C30	121.9 (6)
C16—C11—C12	120.7 (6)	C34—C35—H35	119.0
C16—C11—C10	118.3 (6)	C30—C35—H35	119.0
C12—C11—C10	121.0 (6)	O10—C36—N4	122.9 (6)
O4—C12—C13	119.7 (6)	O10—C36—H36	118.5
O4—C12—C11	125.0 (6)	N4—C36—H36	118.5
C13—C12—C11	115.3 (6)	N4—C37—H37A	109.5
C14—C13—C12	123.3 (6)	N4—C37—H37B	109.5
C14—C13—Br1	117.9 (5)	H37A—C37—H37B	109.5
C12—C13—Br1	118.8 (5)	N4—C37—H37C	109.5
C13—C14—C15	119.5 (6)	H37A—C37—H37C	109.5
C13—C14—H14	120.2	H37B—C37—H37C	109.5
C15—C14—H14	120.2	N4—C38—H38A	109.5
C16—C15—C14	119.3 (6)	N4—C38—H38B	109.5
C16—C15—Br2	121.4 (6)	H38A—C38—H38B	109.5
C14—C15—Br2	119.2 (6)	N4—C38—H38C	109.5
C15—C16—C11	121.8 (7)	H38A—C38—H38C	109.5
C15—C16—H16	119.1	H38B—C38—H38C	109.5

### Hydrogen-bond geometry (Å, °)

**Table:**

*D*—H···*A*	*D*—H	H···*A*	*D*···*A*	*D*—H···*A*
C18—H18C···O7	0.96	2.59	3.364 (9)	137
C37—H37C···O2	0.96	2.48	3.307 (8)	144
O3—H3···O1^i^	0.82	1.98	2.772 (6)	163
O8—H8···O6^ii^	0.82	2.07	2.888 (6)	176
C16—H16···O7^iii^	0.93	2.52	3.422 (9)	163
C29—H29···O2^iv^	0.93	2.45	3.291 (8)	150
C35—H35···O2^iv^	0.93	2.59	3.408 (8)	147

Symmetry codes: (i) −*x*+1, −*y*+2, −*z*; (ii) −*x*, −*y*+1, −*z*+1; (iii) *x*+1, *y*, *z*; (iv) *x*, *y*−1, *z*.
